# Cytokines in the pathogenesis of rheumatoid arthritis: new players and therapeutic targets

**DOI:** 10.1186/s41927-017-0001-8

**Published:** 2017-11-28

**Authors:** Alessia Alunno, Francesco Carubbi, Roberto Giacomelli, Roberto Gerli

**Affiliations:** 1 Rheumatology Unit, Department of Medicine, University of Perugia, Perugia, Italy; 2 Rheumatology Unit, Department of Biotechnological and Applied Clinical Sciences, University of L’Aquila, L’Aquila, Italy; 3 ASL1 Avezzano-L’Aquila-Sulmona, Department of Medicine, L’Aquila, Italy

## Abstract

In recent years, the landscape of pro- and anti-inflammatory cytokines has rapidly expanded with the identification of new members proven to be involved at different extent in the pathogenesis of chronic immune mediated inflammatory diseases including rheumatoid arthritis (RA). The advance of our understanding of mediators involved in the pathogenesis of RA and in consequence, the development of novel targeted therapies is necessary to provide patients not responding to currently available strategies with novel compounds. The aim of this review article is to provide an overview on recently identified cytokines, emphasizing their pathogenic role and therapeutic potential in RA. A systematic literature review was performed to retrieve articles related to every cytokine discussed in the review. In some cases, evidence from animal models and RA patients is already consistent to move forward into drug development. In others, conflicting observation and the paucity of data require further investigations.Forty years after the discovery of IL-1, the landscape of cytokines is continuously expanding with increasing possibilities to develop novel therapeutic strategies in RA.

**Electronic supplementary material:**

The online version of this article (10.1186/s41927-017-0001-8) contains supplementary material, which is available to authorized users.

## Background

Rheumatoid arthritis (RA) is a chronic inflammatory disease characterized by inflammation of the synovial membrane. The release of pro-inflammatory cytokines as well as other pro-inflammatory molecules results in joint destruction and disability [1, 2]. To date, the exact cause of RA has not been identified but several studies pointed out that pro-inflammatory cytokines, including tumor necrosis factor (TNF)-α, interleukin (IL)-1, IL-6, IL-17 and the mediators produced through downstream pathways in the arthritic joints, constitute the milieu driving cartilage and bone destruction [3]. On this basis, therapeutic possibilities for RA patients include monoclonal antibodies, fusion proteins or antagonists against these molecules. However, partial and non-responses to these compounds, together with the increasing clinical drive to remission induction, requires that further therapeutic targets are identified [4]. In recent years, a growing number of new cytokines as well as their function in health and disease have been identified [5]. Cytokines serve as the mediators of cellular differentiation, inflammation, immune pathology, and regulation of the immune response. In particular, novel inflammatory mediators with their associated cell signaling events have now been proven to have a role in experimental arthritis and in RA, including members of the IL-1 (IL-33, IL-36, IL-37, IL-38) and IL-12 (IL-27, IL-35) superfamilies, and other cytokines such as IL-32, IL-34. The aim of this review article is to provide an overview on these recently identified cytokines, emphasizing their pathogenic role and therapeutic potential in RA. Table 1 summarizes all the available data in animal models and RA patients for each cytokine.

**Table 1:** Data on different cytokines in experimental arthritis and patients with rheumatoid arthritis

	Experimental arthritis	Rheumatoid arthritis			
		serum	plasma	SF	ST
IL-12 family					
IL-27	IL-27Rα KO mice develop more severe CIA [21] IL-27 triggers PGIA [22] IL-27 administration ameliorates CIA and AIA [23–25, 27]	↑ vs HD [28] ↑ in RA-ILD [28]	↑ vs HD [29] = vs HD and OA [30]	↑ vs OA [30]	↑ vs OA [30]
IL-35	IL-35 ameliorates CIA [110–112]	↑ in early RA vs established RA [113] ↓ following DMARD therapy ↓ vs HD [115] Inverse correlation with disease activity [115]	na	↑ vs OA [113]	↑ vs OA and PsA[114]
IL-1 family					
IL-33	Development and severity of CIA in IL-33 KO mice is comparable to that of WT mice [67] Mice lacking ST2 develop attenuated CIA and AIA [68, 69] Treatment of WT mice with recombinant (r) IL-33 significantly exacerbated CIA and AIA[68, 69]	↑ vs HD, OA and PsA [70–73] ↑ in RA-ILD [71] ↑ in erosive RA [71] Lower baseline levels predict good response to anti-TNF-α agents [74, 75] Detectable levels at baseline predict response to RTX [77] Detectable levels at baseline predict atherosclerotic plaque progression [76]	na	↑ vs OA [26] = vs OA [72] Lower baseline levels predict good response to anti-TNF-α agents [74, 75]	
IL-36	IL-36 is upregulated in CIA, CAIA and AIA [122, 123] IL-36 blockade does not affect arthritis [122, 123]	↑ vs HD [133]	na		↑ vs OA [126]
IL-37	Systemic and intra-articular administration of recombinant IL-37 inhibits the development of synovitis in CIA and AIA [146, 147]	↑ vs HD [133]	↑ vs HD and OA [147–150] ↑ in FR^+^ and anti-CCP^+^ patients vs seronegative [150] ↑ in active vs inactive RA [149] ↑ in erosive RA [150]	↑ [150]	↑ [147]
IL-38	IL-38 KO mice display more severe AIA [131] IL-38 overexpression attenuates CIA and STIA [132]	= vs HD and OA [131] ↑ vs HD [133]	na		↑ vs OA [131]
Other					
IL-32	IL-32 administration worsens CIA [44]	↑ vs HD and OA [46, 47]	na		↑ vs OA [48, 49]
IL-34	IL-34 KO mice do not display any autoimmune manifestations [87, 88] CSF-1R blockade is associated with less severe mBSA-IA and CIA [93–95]	↑ vs HD, OA, PsA, AS [96–100] Levels correlate with RF, anti-CCP, ESR, CRP, disease activity, smoking [96–99] Baseline levels predict radiographic progression [97, 99]	na	↑ vs HD, OA, PsA, AS [96, 100] Levels are directly correlated with those of SF RANK-L	↑ [95, 101, 102] Expression associated with the severity of synovitis [95, 101, 102]

*SF* synovial fluid, *ST* synovial tissue, *KO* knock-out, *WT* wild type, *CIA* collagen induced arthritis, *PGIA* proteoglycan-induced arthritis, *AIA* antigen induced arthritis, *CAIA* collagen antibody-induced arthritis, *STIA* K/BxN serum transfer-induced arthritis, *mBSA* methylated bovine serum albumin, *HD* healthy donors, *OA* osteoarthritis, *PsA* psoriatic arthritis, *AS* ankylosing spondylitis, *ILD* interstitial lung diseas, TNF, tumour necrosis factor, *RTX* rituximab, *RF* rheumatoid factor, *anti-CCP* anti cyclic cutrullinated peptide, *ESR* erythrosedimentation rate, *CRP* C reactive protein, *RANK-L* receptor activator of nuclear factor κ-B ligand, *DMARDs* disease modifying anti-rheumatic drugs

### New members of IL-1 family

#### IL-33

IL-1 cytokine includes 11 pro-inflammatory and anti-inflammatory members, chronologically named according to their discovery, IL-1 family member 1 (IL-1F1) to IL-1F11. More commonly, they are also known as IL-1α, IL-1β, IL-1 receptor antagonist (IL-1Ra), IL-18, IL-33, IL-36α, IL-36β, IL-36γ, IL-36Ra, IL-37, and IL-38 [6]. All IL-1 cytokines bind to similar receptors consisting of extracellular immunoglobulin domains and intracellular Toll/IL-1 (TIR) domains. The signal is transduced via cytoplasmic myeloid differentiation primary response protein 88 (MyD88) and IL-1R associated kinase 4 (IRAK4), ending up in the activation of transcriptions factors like NF-kB or MAPK [7]. IL-33 (IL-1F11) was identified in high endothelium venules in 2003 [8]. Subsequent studies revealed that IL-33 acts as alarmin, being modulated by inflammatory stimuli. Indeed, IL-33 is up-regulated during the inflammatory response and can be released by necrotic cells. On the other hand, IL-33 is inactivated by caspase-1 during apoptosis [9]. IL-33R ST2 belongs to the family of IL-1R and, upon binding to the ligand, triggers the transduction signal via the NF-kB or MAPK pathways [10]. ST2 is expressed by several immune cells including basophils, mast cells, eosinophils, DCs and NK cells. However, the most important target of IL-33 is represented by Th2 cells. Besides its trans-membrane form, ST2 can be released in a soluble form (sST2) by different immune and non-immune cell types thereby blocking IL-33 effects [11]. Being involved in Th2 immune response, IL-33 has been extensively investigated in the field of allergic diseases [12]. Circulating and tissue levels of IL-33 are increased in experimental models of asthma [13, 14] and blockade of this pathway is able to ameliorate airway inflammation [13, 15–17], thereby confirming an in vivo inhibition of IL-33-mediated effects. With regard to experimental arthritis, the development and severity of CIA in IL-33 KO mice is comparable to that of WT mice [18]. However, mice lacking ST2 develop attenuated CIA and AIA and treatment of WT mice with recombinant (r) IL-33 significantly exacerbated both [19, 20]. Therefore, data from animal models do not provide univocal evidence. In RA patients, IL-33 serum levels are increased compared to normal and disease controls (OA and psoriatic arthritis (PsA)) [21–24]. Evidence from RA SF is conflicting as IL-33 was found to be either increased [25] or comparable [23] to OA SF. Conflicting results were also obtained with regard to the correlation of serum and SF IL-33 in RA paired samples. In fact, both an inverse [21] and a direct association have been reported [25]. Of interest, RA patients with higher active disease display higher levels of this cytokine in serum [21] and SF [26] and higher IL-33 serum levels were also associated to bone erosions and RA-ILD [22]. In this regard, and also in general when measuring cytokines in the serum of RA patients, it should be taken in mind that discrepancies could be due to false measurements caused by heterophilic antibodies [27] or differences in patient population. Serum sST2 levels were found to be increased in RA compared to OA [23]. IL-33, IL-33R ST2 and sST2 expression has been claimed as possible markers of response to treatment in RA. First, RA patients achieving a good response with an anti-TNF-α treatment display lower levels of IL-33 in serum and SF and IL-33R on immune cells compared to patients treated with methotrexate or non-responders to TNF-α inhibitors [28, 29]. This is further supported by the evidence that neutrophils of anti-TNF-α responder patients respond to a lesser extent to IL-33 in vitro compared to methotrexate-treated patients. Secondly, RA patients with lower sST2 levels at baseline are those who more likely achieved remission following 12 months of treatment with disease modifying anti-rheumatic drugs (DMARDs) and anti-TNF-α agents [30]. Finally, baseline detectable serum IL-33 levels have been associate to a good clinical response to rituximab [31]. Interestingly, baseline IL-33 and sST2 levels have been also associated to cardiovascular risk factors. Cardiovascular risk is a severe comorbidity in RA patients being the first cause of death in these patients and persistent inflammation is one of the main determinants. In this regard, baseline level of serum IL-33 is also a predictor for atherosclerotic plaque progression in patients with early RA, independently of other traditional risk factors and other inflammatory biomarkers [30]. Currently available data regarding IL-33 axis in RA do not allow to draw definitive conclusion about its actual role in RA pathogenesis and consequently about its possible therapeutic targeting in this disease.

#### IL-36 and IL-38

The new IL-1 family members IL-36α, IL-36β, IL-36γ, IL-36Ra (IL-1F5) and the antagonist IL-38, bind to the IL-36R consisting of the IL-1 receptor-related protein 2 (IL-1RrP2) and its accessory protein IL-1RAcP. IL-36R is expressed by DCs, CD4^+^ T-cells and macrophages. Binding of the agonists to the membrane bound IL-1RrP2 leads to the recruitment of the co-receptor IL-1RAcP. This triggers an intracellular signaling cascade via JNK, ERK1/2, and NF-kB which results in the production of pro-inflammatory mediators. On the contrary, the binding of the natural inhibitors IL-36Ra and IL-38 prevents the signaling [32, 33]. During the last few years, IL-36 cytokines as well as IL-37 and IL-38 raised growing interest, as they have been involved in various diseases, including RA [34]. IL-37 and IL-38 have been shown to play an anti-inflammatory role in several diseases, whereas IL-36 exerts pro-inflammatory effects. All the three IL-36 agonists induce pro-inflammatory mediators such as cytokines, chemokines and co-stimulatory molecules thereby promoting Th1 and Th17 cell commitment, neutrophil influx and DC activation [35]. In particular, IL-36 and its receptor can stimulate DC and can promote their maturation; DC precursors exposed to IL-36 release IL-12 and contribute to differentiation of T cells to Th1 cells. In vivo, IL-36β can act as an adjuvant to promote Th1 response [36]. In humans, the three IL-36 isoforms and their receptor are over-expressed in psoriasis; moreover, IL-36 can induce the release of pro-inflammatory cytokines, such as IL-6 or IL-8 as well as IL-17, IL-22, and. These cytokines can induce IL-36 release, creating a feedback loop [33, 37]. In mouse models of RA, such as CIA, collagen antibody-induced arthritis (CAIA), and AIA, all of the IL-36 family members are upregulated during acute inflammation. However, treatment with an IL-36R-blocking antibody of TNF-transgenic mice, another experimental model of RA, resulted in no changes in symptoms or clinical onset, suggesting that the severity of experimental arthritis is independent of IL-36R signaling [38, 39]. This evidence can be attributable to the redundancy of IL-1 family member downstream signaling, mainly those of IL-1, which is a major player in experimental arthritis. It is unclear whether this redundancy is similar in human arthritis. Of note, the magnitude of stimulatory effect of IL-36 in synovial fibroblasts and articular chondrocytes was markedly lower than those of IL-1 [40], suggesting that IL-36 is probably not a key player in human arthritis. IL-36β is constitutively expressed in human articular chondrocytes, and stimulation of both synovial fibroblasts and articular chondrocytes by recombinant IL-36β induces proinflammatory cytokine responses [40]. In mice with CIA and in the synovium of patients with RA, IL-36α, IL-36β, IL-36γ, IL-36Ra and IL-38 were all elevated and correlated with IL-1β, CCL3, CCL4 and macrophage (M)-CSF, but not with Th17 cytokines [41]. Expression of IL-36R and its ligands IL-36α and IL-36Ra has been detected in the synovial lining layer and cellular infiltrates of patients with inflammatory arthritis [42]. IL-36α was upregulated in the synovium of patients with PsA and RA, compared to patients with OA. Conversely, IL-36R and its natural antagonist IL-36Ra were expressed at similar levels in the synovial tissue in all three diseases. In the same study, synovial CD138^+^ plasma cells seem to be the main source of IL-36α, and IL-36α is able to induce IL-6 and IL-8 production in synovial fibroblasts [42]. IL-38 (IL-1F10) is released as a 152-amino acid precursor having a molecular weight of 16 kDa. IL-38 shares 41% homology with IL1-Ra and 43% with IL-36Ra [43, 44]. IL-38 binds to IL-36 receptor, as does IL-36Ra, and has similar biological effects on immune cells. Thus, in vitro, IL-38 inhibits the production of Th1 cytokines, IL-17, and IL-22. IL-38, similarly to IL-36Ra, has an anti-inflammatory effect on PBMCs, contrasting with a clearly pro-inflammatory effect on DC with increased IL-6 production [45]. IL-38 is selectively secreted by human apoptotic cells to counteract inflammation. The depletion of IL-38 in apoptotic cells leads to an increase of pro-inflammatory cytokine release by macrophages and to the subsequent expansion of Th17-cell at expense of IL-10-producing T cells [46]. Interestingly, in this study, full length recombinant IL-38 induced IL-6 production by macrophages, whereas truncated IL-38 decreased IL-6 expression after X-linked interleukin-1 receptor accessory protein-like 1 (IL-1RAPL1) binding. However, it is still unclear whether IL-38 is an inflammatory or an anti-inflammatory cytokine. IL-38 seems to have either pro- or anti-inflammatory effects depending on the dose. Whereas IL-38 gene deficiency enhanced arthritis, systemic administration of recombinant IL-38 protein did not inhibit arthritis development. Therefore, it is possible that IL-38 may have dose-dependent effects in inflammatory vs. anti-inflammatory responses. Further analysis is required to test this hypothesis. Takenaka et al. investigated AIA in IL-38 KO and observed greater disease severity, accompanied by higher IL-1β and IL-6 gene expression in the joints compared to control mice [47]. Recently, Boutet et al. demonstrated that adeno-associated virus-mediated IL-38 overexpression exerted moderate but significant anti-inflammatory effects in CIA and K/BxN serum transfer-induced arthritis (STIA) [48]. In addition to the reduced macrophage number, a significant decrease in the expression of Th17 cytokines (IL-17, IL-22), IL-6, TNF-α and CXCL1 was observed in this study, without any modification in IL-1β expression. Of note, IL-38 overexpression did not induce the production of other anti-inflammatory cytokines, but reduced significantly IL-10 expression. IL-38 levels are increased in the synovial membrane and sera from patients with RA compared with healthy controls [47, 49] IL-38 is expressed by keratinocytes, synovial fibroblast from patients with RA, as well as by human monocytes and type I macrophages polarized in vitro [41].

Taken together, data about IL-36 in RA seem to support the pursuit of its blockade for therapeutic purposes in this disease, Conversely, whether the anti-inflammatory cytokine IL-38 should be considered a new therapeutic option in arthritis or other inflammatory diseases deserves further experiments.

#### IL-37

IL-37, previously known as IL-1 family member 7 (IL-1F7), is a member of the IL-1 family initially identified in early 2000s [49]. IL-37b is the largest of the 5 different splice variants (from a to e) and its precursor is cleaved by caspase-1 into mature IL-37b. IL-37 is expressed in several tissues, is associated with plasma cells and it is constitutively expressed in the cytoplasm of monocytes and PBMCs [50]. TLR agonists and pro-inflammatory cytokines, including IL-1β, TNF-α and IFN-γ can upregulate IL-37 in PBMCs [51]. Upon binding to its receptor shared with IL-18, IL-18R, IL-37 is able to inhibit the transcription of several pro-inflammatory cytokines, including IL-17, via the suppression of the MAPK pathway [52, 53]. Recent data demonstrated that IL-37 also needs IL-1R8, a member of the IL-1R family, to exert such anti-inflammatory activity. In fact, transgenic mice overexpressing IL-37 are protected from lipopolysaccharide (LPS)-induced shock [53], but only if the IL-1R receptor is correctly expressed in order to form the tripartite IL-37–IL-1R8–IL-18Rα complex on the surface of PBMCs upon stimulation with LPS [54]. Similarly, the administration of recombinant IL-37 consistently reduced LPS-induced inflammation [54]. In addition, IL-37 transgenic mice develop less severe colitis and psoriasis [55, 56]. Based on the anti-inflammatory activity of IL-37, several studies have been performed to investigate whether the administration of IL-37 may ameliorate chronic inflammation [57]. Studies performed in patients reported an overall increase of circulating as well as tissue IL-37 in inflammatory bowel disease [58], systemic lupus erythematosus [59], Graves’ disease [60] and AS [61]. With regard to experimental arthritis, systemic and intra-articular administration of recombinant IL-37 was able to inhibit the development of synovitis by reducing pro-inflammatory cytokines and modulating Th17 cells [62, 63]. In striking contrast with experimental RA, but in line with the results obtained in patients with other autoimmune disease, studies performed in RA patients revealed higher serum and plasma levels of IL-37 compared to normal and OA controls [63–66]. Such increase is particularly evident in patients with active disease, compared to patients in remission [65], and in patients with positive RF and anti-CCP [66]. Furthermore, IL-37 levels have been correlated to pro- and anti-inflammatory cytokines (IL-4, IL-7, IL-10, IL-12, IL-13, IL-17A, TNF-α) as well as to disease activity and bone erosions. Moreover, they are reduced by DMARD and anti-TNF-α treatment in patients with a good clinical response [64–66]. The only available study assessing IL-37 levels in RA SF reported increased levels of this cytokine compared to paired serum samples [66]. IL-37 is also consistently expressed in the synovial tissue of RA patients with active disease [63]. These findings may be explained, at least in part, by the evidence that IL-37 plasma concentration in RA is directly correlated with pro-inflammatory cytokines, including IL-17 and TNF, as well as with disease activity and radiographic bone erosion score and bone loss [64, 67]. It is therefore reasonable to speculate that such increase of IL-37 may be a compensatory mechanism to counteract the effector immune response, likely occurring also in other autoimmune diseases. This mechanism is not effective of course either because IL-37 levels are insufficient or because the cytokine is neutralized by factors that need to be elucidated.

Based on this, the potentiation of IL-37 as well the identification of its agonists may represent an intriguing approach for therapeutic purposes in RA.

### New members of IL-12 family

#### IL-27

IL-27 is a newly identified heterodimeric cytokine belonging to the IL-12 family, which includes IL-12, IL-23, IL-27, and IL-35 [68]. The IL-12 cytokine family is part of the IL-6 superfamily of type I cytokines. However, while IL-6 family members are secreted as single-subunit monomers, those of the the IL-12 family are heterodimeric. The four members of the IL-12 cytokine family are consisted of an α chain (p19, p28, or p35) and a β chain (p40 or Epstein-Barr virus induced gene 3 (EBI3)) [69]. In detail, p35 and p40 subunits constitute IL-12, p19 and p40 subunits constitute IL-23 and p28 combines with EBI3 forming IL-27. The latest recognized member, IL-35, consists of p35 and EBI3. For the sake of completeness, it should be mentioned that very recently another family member, IL-39, has been described. IL-39 is composed of IL-23p19 and EBI3 heterodimer, is secreted by activated B lymphocytes and seems to play a pathogenic role in mouse models of systemic lupus erythematosus [70, 71], but no data on RA or other diseases are available so far. These cytokines transduce the signal through unique pairings of 5 receptor chains: IL-12Rβ1, IL-12Rβ2, IL-23R, IL-27Rα (or WSX-1) and gp130. IL-12 signals through IL-12Rβ1 and IL-12Rβ2, IL-23 signals through IL-23R and IL-12Rβ1, and IL-27 signals through gp130 and IL-27Rα [72]. In T cells, IL-35 signals through IL-12Rβ2 and gp130, although it can also signal through IL-12Rβ2/IL-12Rβ2 and gp130/gp130 homodimers [73]. Signal transduction through these receptor chains is mediated by the Janus kinase (JAK)-signal transducer and activator of transcription (STAT) pathway. Despite similarities in the structural cytokine subunits, receptor components, and downstream signaling, IL-12 family members display diverse but balanced functions. IL-12 and IL-23 represent the strictly pro-inflammatory members with key roles in T helper (h) 1 and Th17 development [26, 74], while IL-27 carries out its role in inflammation supporting Th1 development and interferon (IFN)-γ production and inhibiting Th2 and Th17 differentiation programs [75, 76]. IL-27 is mainly produced by antigen presenting cells (APCs), including dendritic cells (DCs) and macrophages, as well as by endothelial cells. Besides the JAK/STAT pathway, IL-27 can also activate other pathways including p38- mitogen-activated protein kinase (MAPK) and AKT in specific cell types, such as liver and intestinal epithelial cells [77]. gp130 is ubiquitously express in a wide range of cell types including mast cells and natural killer (NK) cells while WSX-1 is consistently expressed by naïve T cells and to a greater extent by activated and memory cells, the latter being therefore highly susceptible to the effects of this cytokine [78]. Indeed, IL-27 is a pivotal cytokine in the commitment of naïve T cells. It was first described as a Th1-polarizing cytokine being able to induce expression of T-bet and suppression of GATA-3 [77]. However, the evidence that IL-27R knockout (KO) mice develop a hyper-inflammatory phenotype, prompted to explore possible effects of this cytokine on other T-cell subsets. The subsequent demonstration that IL-27 can both increase the secretion of IL-10 by naïve T cells, thereby inducing regulatory T (Treg) cells, and directly antagonize the development of pro-inflammatory Th17-cell responses, allowed to speculate that IL-27 may have a protective role in chronic immune mediated inflammatory diseases [79]. Interestingly, studies evaluating the contribution of each receptor subunit function revealed that the lack of WSX alone does not affect the anti-inflammatory properties of IL-27 [80, 81]. With regard to RA animal models, collagen induced arthritis (CIA) in IL-27Rα KO mice is characterized by a more severe clinical picture with synovial germinal center -like structures, increased leukocyte infiltration, synovial hypertrophy, and cartilage/bone erosion compared to wild type (WT) mice [82]. Conversely, in proteoglycan-induced arthritis (PGIA), IL-27 seems to be crucial to trigger the inflammatory response [83]. Therefore, although the majority of studies agree that both systemic and local administration of IL-27 ameliorates CIA and adjuvant induced arthritis (AIA) [84–87], still the negative effect observed in PGIA requires additional evaluation. With regard to the human counterpart, serum IL-27 levels are increased in RA and seem to be directly correlated with disease activity and RA-associated interstitial lung disease (ILD) [88]. Wong et al. reported that IL-27 is higher in RA plasma compared to normal subjects [89], while Tanida et al. failed to observe any difference between RA, osteoarthritis (OA) and healthy controls [90]. Of interest, however, the latter study revealed that IL-27 is highly expressed in RA synovial fluid (SF) and synovial tissue. Synovial IL-27 mainly derives from CD14^+^ mononuclear cells (MNC) rather than from fibroblast-like synoviocytes (FLS). These IL-27 producing CD14^+^ MNCs are virtually absent in OA synovium where IL-27 is barely detectable. The production of pro-inflammatory cytokines and chemokines including IL-6 by RA-FLS in vitro is inhibited by IL-27. Therefore, it may be hypothesized that IL-27 exerts, or at least attempts to, an anti-inflammatory effect in RA synovial environment via the inhibition of Th17-cell commitment. In light of the well-established role of Th17 cells in RA pathogenesis, and in particular in the development of synovial germinal center-like structures, Jones et al. demonstrated that synovial IL-27 expression is more pronounced in germinal center-negative RA synovium compared to germinal center-positive RA synovium and OA synovium, and that it is inversely correlated to the expression of molecules involved in ectopic lymphoid neogenesis. These findings, are in line with those obtained in experimental RA and allow to speculate that IL-27 may have a protective role in this disease [82].

Currently available data about IL-27 highlight that this is another cytokine owing an anti-inflammatory activity and therefore the identification of molecules acting as IL-27 agonists may represent an intriguing option to be explored in RA.

#### IL-35

IL-35 is a heterodimeric cytokine belonging to the IL-12 family together with IL-12, IL-23, IL-27 and IL-35 [91]. In T cells, IL-35 signals through IL-12Rβ2 and gp130, although it can also signal through IL-12Rβ2/IL-12Rβ2 and gp130/gp130 homodimers [92]. Interestingly, although all the receptors for IL-35 induce suppression of T-cell proliferation, the homodimeric receptors are unable to mediate the generation of IL-35 induced regulatory T cells (iTr35) [92]. IL-35 signals both through gp130 and IL-12Rb2 homodimers and through an IL-12Rb2:gp130 heterodimeric receptor but only the latter can mediate T-cell suppression and iTr35 induction through the formation of pSTAT1:pSTAT4 heterodimers [92]. Furthermore, IL-35 signaling through an IL-12Rβ2:WSX-1 heterodimer and the induction of pSTAT1 and pSTAT3 is peculiar of B cells [93]. IL-35 is mainly released by Treg cells, is required to potentiate the suppressive activity of murine and human Treg cells and therefore inhibits T-cell proliferation in vitro and in vivo disease models [94]. Recently, it has been reported that also regulatory B (Breg) cells can produce IL-35 [93, 95]. Finally, as shown for IL-10 and transforming growth factor (TGF)-β, IL-35 can also induce the conversion of naïve T cells into iTr35 cells [96]. The CIA mouse model shares many similarities with human RA as synovial cells proliferate in a tumor-like manner and cause synovitis. Angiogenesis is a shared pathogenic process, hence vascular endothelial growth factor (VEGF) is a crucial player in tissue injury/repair, inflammation and eventually in RA development [97]. IL-35 plays an anti-inflammatory role by inducing Treg-cells and inhibiting Th17 cell commitment in several experimental models of inflammatory diseases including CIA [98]. Moreover, IL-35 treatment inhibited proliferation and promoted apoptosis in cultured FLS from CIA mice in a dose-dependent manner [99]. Finally, IL-35 seems to inhibit angiogenesis of CIA mice as well as downregulate the expression of VEGF and its receptors, ameliorating the severity of synovitis [100]. Unfortunately, data on IL-35 in patients affected by RA remain controversial. In particular, while some Authors support anti-inflammatory activities of IL-35, others suggest its pro-inflammatory properties. IL-35 was found to be higher in patients with treatment naïve early RA compared to those with established disease and to be reduced after 3 months treatment with glucocorticoids and conventional synthetic (cs) DMARDs [101]. IL-35 was found to be also higher in SF RA compared to PsA and control OA patients and correlated with higher disease activity, supporting a potential role of IL-35 in the pathogenesis of RA [101, 102]. Moreover, TNF-α can induce the expression of both p35 and EBI3 subunits in FLS and MCs, and since the latter express both subunits of IL-35 receptor can secrete several pro-inflammatory molecules (IL-1β, IL-6 and MCP-1) upon IL-35 stimulation [102]. Nakano et al. reported that serum levels of IL-35 are decreased in RA patients, when compared with normal controls, mainly in patients with active disease, with an inverse correlation between serum IL-35 levels and the 28-joint disease activity score (DAS) based on CRP [103]. The function of IL-35 was also evaluated in a suppression assay using T cells isolated from human RA patients; recombinant IL-35 facilitated the function of natural Treg cells in vitro and restrained pro-inflammatory cytokines such as IL-17 and IFN-γ [103]. These conflicting results may be explained at least in part by the heterogeneity of patient cohorts, also from a genetic point of view, and different disease activity scoring systems. Further studies, especially in larger cohorts of patients are required to clearly explore the immunosuppressive role and potential therapeutic benefits of targeting IL-35 in RA.

#### IL-32

IL-32 is a cytokine produced by immune and non-immune cells, and has recently gained popularity because of its important biological functions [104]. IL-32 gene was found to be located on human chromosome 16p13.3 and was reported to exist in nine different isoforms by mRNA alternative splicing including IL-32훼, IL-32훽, IL-32훾, IL-32훿, IL-32휀, IL-32휁, IL-32휂, IL-32휃, and IL-32 s (small), with specific activities and properties. Moreover, these isoforms can interact with each other intracellularly to control their respective activities and IL-32훾 is the most active isoform [105–107]. IL-32 is not assigned to any of the cytokine families, due to the lack of homology with other well-known cytokines. IL-32 was originally described as an mRNA called NK cell transcript 4 (NK4), which encoded a protein with many characteristics of a cytokine, derived from IL-2 activated natural killer cells [108]. NK cells, monocytes/macrophages, T lymphocytes, as well as epithelial cells, endothelial cells, fibroblasts, and hepatocytes, express IL-32 [109], mainly intracellularly, although some reports suggest that the IL-32γ isoform, could be secreted in limited amounts [110]. However, depending on the cell type and stimulus, IL-32 may be released after necrotic cell death or in vesicles such as exosomes [111, 112]. One problem that remains associated with IL-32 is the identification of cell surface receptor of IL-32. IL-32 is a pleiotropic cytokine and an important player in innate and adaptive immune responses, involved in a number of biological functions, including cell differentiation, stimulation of pro- or anti-inflammatory cytokines and cell death, especially apoptosis [113]. In detail, this cytokine induces other pro-inflammatory cytokines and chemokines such as TNF-α, IL-1β, IL-6, and IL-8 by means of the activation of NF-kB and p38-MAPK. IL-32, via caspase-3 activity, induces differentiation of monocytes into macrophage-like cells with characteristics of generating pro-inflammatory cytokines such as IL-6, TNFα and chemokines [114]. IL-32γ, via a phospholipase C (PLC)/ c-Jun N-terminal kinases (JNK)/NF-kB-dependent pathway, induces maturation and activation of DCs, leading to increased production of IL-12 and IL-6, Th1- and Th17-polarizing cytokines [115]. Moreover, IL-32 synergizes with nucleotide oligomerization domain (NOD) 1 and NOD2 ligands for IL-1β and IL-6 production, through a caspase 1-dependent mechanism [116]. Finally, IL-32β, increasing adhesion of inflammatory cells to activated endothelial cells with consequent induction of pro-inflammatory cytokines, is involved in the propagation of vascular inflammation [117]. Its production is predominantly induced by IL-1β, TNF-α, IL-2 or IFN-γ in blood monocytes and epithelial cells [39]. In addition to cytokines, microbial products, including viruses, have emerged as potent inducers of IL-32 in human monocytes, macrophages, and monocyte-derived DCs [109]. All the above mentioned data clearly point out that IL-32 and TNF-α are strongly linked to each other and being TNF-α a key cytokine in RA pathogenesis, IL-32 may play profound effects in this process [118]. Studies from animal models demonstrated that human IL-32, when injected in joints of naïve mice, leads to increased expression of inflammatory molecules (IL-1β, TNF-α, IL-18, IFN-γ, IL-17, IL-21 and IL-23), recruitment of inflammatory cells, cartilage derangements and joint swelling [119]. Conversely, joint swelling and presence of inflammatory cells drastically decreased in a TNF-α deficient mouse model [120]. This observation further supports the key interplay between TNF-α and IL-32 in RA pathogenesis. Furthermore, the unmasking of the molecular mechanism of the IL-32/ TNF-α in RA open new avenues for their potential therapeutic targeting. Several studies confirmed an overexpression of IL-32 and IL32γ in RA patients, when compared to osteoarthritis or healthy volunteers [121, 122]. In particular, the IL-32γ level was found significantly upregulated in CD14^+^ monocytes and synovial membrane of RA patients [123, 124]. High levels of IL-32 in synovial biopsies of RA, as compared to its absence in OA patients, suggested that IL-32 is potent mediator of active osteoclastogenic activity. In particular, the synergism between IL-32 and soluble receptor activator of nuclear factor κ-B ligand (sRANK-L) enhances the activity of osteoclasts and consequently tissue resorption [124]. Both IL-32 and IL-17 can reciprocally influence each other’s production and amplify the function of osteoclastogenesis in RA synovium [124]. RA FLS seem to have a key role in osteoclastic activity, as well as in pannus formation in the joint [125]. IL-32β, δ, and γ mRNA overexpression in RA FLS is primarily induced by TNF-α, IFN-γ and toll-like receptor (TLR)-2, −3, and −4 ligands, and the overexpression of IL-32 seems to stabilize the mRNA transcripts of other cytokines, in particular TNF-α, IL-1β and IL-8 [110, 126]. In FLS, TNF-α-activates Syk/PKC-d/JNK/c-Jun pathway to induce IL-32 (isoforms α, β, δ, and γ) [127], suggesting a splicing of IL-32γ into IL-32β [110]; interestingly, IL-32β is associated with lower inflammation and less severity of RA when compared with IL-32γ. IL-32 stimulates the synthesis of prostaglandin E2, an important mediator of cartilage and bone destruction in RA [128]. Very few clinical data regarding IL-32 response in patients treated with anti-TNF-α therapy are available; in particular, synovial knee biopsies showed a significant decrease in IL-32 expression in RA patients treated with a TNF-α blocker [129]. This observation fits with the evidence of a direct correlation between IL-32, TNF-α and disease activity in RA [130]. Additional studies, especially in human systems, are necessary to resolve the inconsistency of IL-32 in RA as well as to explore the therapeutic potential of this cytokine in RA.

#### IL-34

IL-34 has been discovered in 2008 [131] and the receptor to which IL-34 binds with the highest affinity, colony stimulating factor (CSF)-1R, is shared with CSF-1. However, IL-34 and CSF-1 do not share sequence homology and have different expression patterns being IL-34 restricted to few tissues (brain, epidermis, spleen, bone marrow, lymph nodes) and CSF-1 widespread [132]. Upon binding to CSF-1R, IL-34 stimulates monocytes and macrophages through extracellular signal-regulated kinase (ERK) 1/2 or AKT phosphorylation. Recent data, however, demonstrated that IL-34 could also bind chondroitin sulphate chains, such as PTP-ζ and syndecan-1, but with lower affinity [133, 134]. This is of particular importance in tumor biology as these receptors are up-regulated in several cancer types. IL-34 can be induced by a variety of pro-inflammatory cytokines, including IL-1β and TNF-α, and its main function of that to promote monocyte survival, proliferation and differentiation to macrophages. Recent studies revealed that IL-34 drives the differentiation of monocytes into immunosuppressive M2 and that human macrophages cultured in the presence of IL-34 are able to expand Treg cells. Interestingly, IL-34-expanded Treg cells display a stronger suppressive activity compared to non–IL-34–expanded Treg cells [135, 136]. This widens the spectrum of action of IL-34 towards immune tolerance. Moreover, IL-34 is involved in RANK-L mediated osteoclastogenesis by inducing the proliferation and adhesion of osteoclast progenitors in vitro and by inducing the formation of osteoclasts from murine splenocytes in vivo, thereby reducing trabecular bone mass [137–139]. IL-34 deficient mice selectively lack Langerhans cells and microglia and display weak immune responses to skin antigens and central nervous system-selective viruses, but they display neither osteopetrosis nor any autoimmune manifestation [140, 141]. Mice lacking CSF-1R receptor are toothless and severely osteopetrotic and display circulating monocyte depletion, total depletion of microglia, significant impairment of olfactory function, defects in reproductive function and reduced bone marrow hematopoietic progenitor cells [142–144]. Conversely, the neutralization of CSF-1R in adult mice leads to a reduction of mature monocytes in blood and bone marrow, without affecting precursors [145]. With regard to experimental arthritis, it is interesting to note that the lack/blockade of CSF-1 as well as the blockade of CSF-1R is associated with less severe methylated bovine serum albumin (mBSA)-induced arthritis and CIA [146–148]. In RA patients, all available studies pointed to increased serum and SF levels of IL-34 with respect to normal and disease controls (OA, PsA, ankylosing spondylitis (AS)) [149–153]. Of interest, ser0075m IL-34 levels correlated with immunological markers of more severe disease including rheumatoid factor (RF), anticyclic citrullinated peptide antibody (anti-CCP) titers, erythrocyte sedimentation rate (ESR), C-reactive protein (CRP), and with disease activity and smoking [149–152]. In this regard, serum IL-34 levels have been also associated with radiographic progression and appear to be good predictors of radiographic damage in RA patients [150, 152]. Interestingly, treatment with DMARDs or TNF-α inhibitors is able to reduce serum IL-34 levels [151, 154]. IL-34 levels are also higher in RA SF compared to OA SF and increased in RA patients with higher disease activity [149, 153]. Of interest, SF IL-34 levels are directly correlated with those of SF RANK-L, further supporting the link between IL-34 and RANK-L mediated osteoclastogenesis [149]. Finally, IL-34 is also consistently expressed in RA ST, mainly in the sublining and the intimal lining layer, with its expression being associated to synovitis severity [148, 154, 155]. All these observations about IL-34 raise the question whether the blockade of its pro-inflammatory and bone remodeling effects are worth the loss also of the strongly suppressive IL-34 driven Treg cells. Therefore additional data are needed to clarify its therapeutic potential in RA.

## Conclusion

The progression and severity of inflammation in RA is associated with a consistent production of pro-inflammatory cytokines and a deregulation of anti-inflammatory cytokines. Although several biologic agents with different mechanisms of action are available for the treatment of RA, even now a consistent number of patients either do not respond or respond only partially to these compounds. Therefore, the advance of our understanding of mediators involved in the pathogenesis of RA and in consequence, the development of novel targeted therapies, are compelling. Forty years after the discovery of IL-1, the never-ending quest to identify ‘the’ culprit of RA development is still a fascinating field under intense investigation. In recent years, the landscape of pro- and anti-inflammatory cytokines has rapidly expanded with the identification of new members proven to be involved at different extent in the pathogenesis of RA. In some cases, evidence from animal models and RA patients is already consistent to move forward into drug development. In others, conflicting observation and the paucity of data require further investigations.

## Additional file

Additional file 1: Reviewer reports and AU response to reviewers. (DOCX 17 kb)

## Abbreviations

AIA: Adjuvant induced arthritis
anti-CCP: Anticyclic citrullinated peptide antibody
APCs: Antigen presenting cells
AS: Ankylosing spondilytis
Breg: Regulatory B
CAIA: Collagen antibody-induced arthritis
CIA: Collagen induced arthritis
CRP: C-reactive protein
CSF: Colony stimulating factor
DC: Dendritic cell
DMARDs: Disease modifying anti-rheumatic drugs
EBI3: Epstein-Barr virus induced gene 3
ERK: Extracellular signal-regulated kinase
ESR: Erythrocyte sedimentation rate
FLS: Fibroblast-like synoviocytes
helper: h
IL-1RAPL1: X-linked interleukin-1 receptor accessory protein-like 1
ILD: Interstitial lung disease
interleukin: IL
IRAK4: IL-1R associated kinase 4
iTr35: IL-35-induced regulatory T cells
JAK: Janus kinase
KO: Knockout
LPS: Lipopolysaccharide
mBSA: Methylated bovine serum albumin
MC: Mononuclear cells
MyD88: Myeloid differentiation primary response protein 88
NK: Natural killer
NK4: NK cell transcript 4
NOD: Nucleotide oligomerization domain
OA: Osteoarthritis
PB: Peripheral blood
PGIA: Proteoglycan-induced arthritis
PsA: Psoriatic arthritis
R: Receptor
RA: Rheumatoid arthritis
RANK-L: Nuclear factor κ-B ligand
RF: Rheumatoid factor
SF: Synovial fluid
STAT: Signal transducer and activator of transcription
TGF: Transforming growth factor
TIR: Intracellular Toll/IL-1
TLR: Toll-like receptor
TNF: Tumor necrosis factor
Treg: Regulatory T
VEGF: Vascular endothelial growth factor
WT: Wild type

## References

[1] Smolen JS, Aletaha D, McInnes IB (2016). Rheumatoid arthritis. Lancet.

[2] Picerno V, Ferro F, Adinolfi A, Valentini E, Tani C, Alunno A (2015). One year in review: the pathogenesis of rheumatoid arthritis. Clin Exp Rheumatol.

[3] Alghasham A, Rasheed Z (2014). Therapeutic targets for rheumatoid arthritis: progress and promises. Autoimmunity.

[4] Moots RJ, Naisbett-Groet B (2012). The efficacy of biologic agents in patients with rheumatoid arthritis and an inadequate response to tumour necrosis factor inhibitors: a systematic review. Rheumatology (Oxford).

[5] Burska A, Boissinot M, Ponchel F (2014). Cytokines as biomarkers in rheumatoid arthritis. Mediat Inflamm.

[6] Dinarello C, Arend W, Sims J (2010). IL-1 family nomenclature. Nat Immunol.

[7] O’Neill LA (2008). The interleukin-1 receptor/toll-like receptor superfamily: 10 years of progress. Immunol Rev.

[8] Baekkevold ES, Roussigné M, Yamanaka T, Johansen FE, Jahnsen FL, Amalric F, Brandtzaeg P, Erard M, Haraldsen G, Girard JP (2003). Molecular characterization of NF-HEV, a nuclear factor preferentially expressed in human high endothelial venules. Am J Pathol.

[9] Cayrol C, Girard JP (2009). The IL-1-like cytokine IL-33 is inactivated after maturation by caspase-1. Proc Natl Acad Sci U S A.

[10] Schmitz J, Owyang A, Oldham E, Song Y, Murphy E, McClanahan TK, Zurawski G, Moshrefi M, Qin J, Li X, Gorman DM, Bazan JF, Kastelein RA (2005). IL-33, an interleukin-1-like cytokine that signals via the IL-1 receptor-related protein ST2 and induces T helper type 2-associated cytokines. Immunity.

[11] Mildner M, Storka A, Lichtenauer M, Mlitz V, Ghannadan M, Hoetzenecker K, Nickl S, Dome B, Tschachler E, Ankersmit HJ (2010). Primary sources and immunological prerequisites for sST2 secretion in humans. Cardiovasc Res.

[12] Saluja R, Khan M, Church MK, Maurer M (2015). The role of IL-33 and mast cells in allergy and inflammation. Clin Transl Allergy.

[13] Oshikawa K, Yanagisawa K, Tominaga S, Sugiyama Y (2002). Expression and function of the ST2 gene in a murine model of allergic airway inflammation. Clin Exp Allergy.

[14] Hayakawa H, Hayakawa M, Kume A, Tominaga S (2007). Soluble ST2 blocks interleukin-33 signaling in allergic airway inflammation. J Biol Chem.

[15] Coyle AJ, Lloyd C, Tian J, Nguyen T, Erikkson C, Wang L, Ottoson P, Persson P, Delaney T, Lehar S, Lin S, Poisson L, Meisel C, Kamradt T, Bjerke T, Levinson D, Gutierrez-Ramos JC (1999). Crucial role of the interleukin 1 receptor family member T1/ST2 in T helper cell type 2-mediated lung mucosal immune responses. J Exp Med.

[16] Kearley J, Buckland KF, Mathie SA, Lloyd CM (2009). Resolution of allergic inflammation and airway hyperreactivity is dependent upon disruption of the T1/ST2-IL-33 pathway. Am J Respir Crit Care Med.

[17] Liu X, Li M, Wu Y, Zhou Y, Zeng L, Huang T (2009). Anti-IL-33 antibody treatment inhibits airway inflammation in a murine model of allergic asthma. Biochem Biophys Res Commun.

[18] Athari SK, Poirier E, Biton J, Semerano L, Hervé R, Raffaillac A, Lemeiter D, Herbelin A, Girard JP, Caux F, Boissier MC, Bessis N (2016). Collagen-induced arthritis and imiquimod-induced psoriasis develop independently of interleukin-33. Arthritis Res Ther.

[19] Xu D, Jiang HR, Kewin P, Li Y, Mu R, Fraser AR, Pitman N, Kurowska-Stolarska M, McKenzie AN, McInnes IB, Liew FY (2008). IL-33 exacerbates antigen-induced arthritis by activating mast cells. Proc Natl Acad Sci U S A.

[20] Xu D, Jiang HR, Li Y, Pushparaj PN, Kurowska-Stolarska M, Leung BP, Mu R, Tay HK, McKenzie AN, McInnes IB, Melendez AJ, Liew FY (2010). IL-33 exacerbates autoantibody-induced arthritis. J Immunol.

[21] Matsuyama Y, Okazaki H, Tamemoto H, Kimura H, Kamata Y, Nagatani K, Nagashima T, Hayakawa M, Iwamoto M, Yoshio T, Tominaga S, Minota S (2010). Increased levels of interleukin 33 in sera and synovial fluid from patients with active rheumatoid arthritis. J Rheumatol.

[22] Xiangyang Z, Lutian Y, Lin Z, Liping X, Hui S, Jing L (2012). Increased levels of interleukin-33 associated with bone erosion and interstitial lung diseases in patients with rheumatoid arthritis. Cytokine.

[23] Talabot-Ayer D, McKee T, Gindre P, Bas S, Baeten DL, Gabay C, Palmer G (2012). Distinct serum and synovial fluid interleukin (IL)-33 levels in rheumatoid arthritis, psoriatic arthritis and osteoarthritis. Joint Bone Spine.

[24] Hong YS, Moon SJ, Joo YB, Jeon CH, Cho ML, Ju JH, Oh HJ, Heo YJ, Park SH, Kim HY, Min JK (2011). Measurement of interleukin-33 (IL-33) and IL-33 receptors (sST2 and ST2L) in patients with rheumatoid arthritis. J Korean Med Sci.

[25] Tang S, Huang H, Hu F, Zhou W, Guo J, Jiang H, Mu R, Li Z (2013). Increased IL-33 in synovial fluid and paired serum is associated with disease activity and autoantibodies in rheumatoid arthritis. Clin Dev Immunol.

[26] Hunter CA (2005). New IL-12-family members: IL-23 and IL-27, cytokines with divergent functions. Nat Rev Immunol.

[27] Bartels EM, Ribel-Madsen S (2013). Cytokine measurements and possible interference from heterophilic antibodies-problems and solutions experienced with rheumatoid factor. Methods.

[28] Verri WA, Souto FO, Vieira SM, Almeida SC, Fukada SY, Xu D, Alves-Filho JC, Cunha TM, Guerrero AT, Mattos-Guimaraes RB, Oliveira FR, Teixeira MM, Silva JS, McInnes IB, Ferreira SH, Louzada-Junior P, Liew FY, Cunha FQ (2010). IL-33 induces neutrophil migration in rheumatoid arthritis and is a target of anti-TNF therapy. Ann Rheum Dis.

[29] Matsuyama Y, Okazaki H, Hoshino M, Onishi S, Kamata Y, Nagatani K, Nagashima T, Iwamoto M, Yoshio T, Ohto-Ozaki H, Tamemoto H, Komine M, Sekiya H, Tominaga S, Minota S (2012). Sustained elevation of interleukin-33 in sera and synovial fluids from patients with rheumatoid arthritis non-responsive to anti-tumor necrosis factor: possible association with persistent IL-1β signaling and a poor clinical response. Rheumatol Int.

[30] Shen J, Shang Q, Wong CK, Li EK, Wang S, Li RJ, Lee KL, Leung YY, Ying KY, Yim CW, Kun EW, Leung MH, Li M, Li TK, Zhu TY, Yu SL, Kuan WP, Yu CM, Tam LS (2015). IL-33 and soluble ST2 levels as novel predictors for remission and progression of carotid plaque in early rheumatoid arthritis: a prospective study. Semin Arthritis Rheum.

[31] Sellam J, Rivière E, Courties A, Rouzaire PO, Tolusso B, Vital EM, Emery P, Ferraciolli G, Soubrier M, Ly B, Hendel Chavez H, Taoufik Y, Dougados M, Mariette X (2016). Serum IL-33, a new marker predicting response to rituximab in rheumatoid arthritis. Arthritis Res Ther.

[32] Sims JE, Smith DE (2010). The IL-1 family: regulators of immunity. Nat Rev Immunol.

[33] Towne JE, Garka KE, Renshaw BR, Virca GD, Sims JE (2004). Interleukin (IL)-1F6, IL-1F8, and IL-1F9 signal through IL-1Rrp2 and IL-1RAcP to activate the pathway leading to NF-kappaB and MAPKs. J Biol Chem.

[34] Hahn M, Frey S, Hueber AJ (2017). The novel interleukin-1 cytokine family members in inflammatory diseases. Curr Opin Rheumatol.

[35] Gabay C, Towne JE (2015). Regulation and function of interleukin-36 cytokines in homeostasis and pathological conditions. J Leukoc Biol.

[36] Vigne S, Palmer G, Lamacchia C, Martin P, Talabot-Ayer D, Rodriguez E, Ronchi F, Sallusto F, Dinh H, Sims JE, Gabay C (2011). IL-36R ligands are potent regulators of dendritic and T cells. Blood.

[37] Marrakchi S, Guigue P, Renshaw BR, Puel A, Pei XY, Fraitag S, Zribi J, Bal E, Cluzeau C, Chrabieh M, Towne JE, Douangpanya J, Pons C, Mansour S, Serre V, Makni H, Mahfoudh N, Fakhfakh F, Bodemer C, Feingold J, Hadj-Rabia S, Favre M, Genin E, Sahbatou M, Munnich A, Casanova JL, Sims JE, Turki H, Bachelez H, Smahi A (2011). Interleukin-36-receptor antagonist deficiency and generalized pustular psoriasis. N Engl J Med.

[38] Derer A, Groetsch B, Harre U, Böhm C, Towne J, Schett G, Frey S, Hueber AJ (2014). Blockade of IL-36 receptor signaling does not prevent from TNF-induced arthritis. PLoS One.

[39] Lamacchia C, Palmer G, Rodriguez E, Martin P, Vigne S, Seemayer CA, Talabot-Ayer D, Towne JE, Gabay C (2013). The severity of experimental arthritis is independent of IL-36 receptor signaling. Arthritis Res Ther.

[40] Magne D, Palmer G, Barton JL, Mézin F, Talabot-Ayer D, Bas S, Duffy T, Noger M, Guerne PA, Nicklin MJ, Gabay C (2006). The new IL-1 family member IL-1F8 stimulates production of inflammatory mediators by synovial fibroblasts and articular chondrocytes. Arthritis Res Ther.

[41] Boutet MA, Bart G, Penhoat M, Amiaud J, Brulin B, Charrier C, Morel F, Lecron JC, Rolli-Derkinderen M, Bourreille A, Vigne S, Gabay C, Palmer G, Le Goff B, Blanchard F. Distinct expression of interleukin (IL)-36alpha, beta and gamma, their antagonist IL-36Ra and IL-38 in psoriasis, rheumatoid arthritis and Crohn’s disease. Clin Exp Immunol 2016;184:159-173. 10.1111/cei.12761 PMC4837235 26701127

[42] Frey S, Derer A, Messbacher ME, Baeten DL, Bugatti S, Montecucco C, Schett G, Hueber AJ (2013). The novel cytokine interleukin-36alpha is expressed in psoriatic and rheumatoid arthritis synovium. Ann Rheum Dis.

[43] Lin H, Ho AS, Haley-Vicente D, Zhang J, Bernal-Fussell J, Pace AM, Hansen D, Schweighofer K, Mize NK, Ford JE (2001). Cloning and characterization of IL-1HY2, a novel interleukin-1 family member. J Biol Chem.

[44] Bensen JT, Dawson PA, Mychaleckyj JC, Bowden DW (2001). Identification of a novel human cytokine gene in the interleukin gene cluster on chromosome 2q12-14. J Interf Cytokine Res.

[45] van de Veerdonk FL, Stoeckman AK, Wu G, Boeckermann AN, Azam T, Netea MG, Joosten LA, van der Meer JW, Hao R, Kalabokis V, Dinarello CA. IL-38 binds to the IL-36 receptor and has biological effects on immune cells similar to IL-36 receptor antagonist. Proc Natl Acad Sci U S A 2012;109:3001-3005. 10.1073/pnas.1121534109 PMC3286950 22315422

[46] Mora J, Schlemmer A, Wittig I, Richter F, Putyrski M, Frank AC, Han Y, Jung M, Ernst A, Weigert A, Brüne B (2016). Interleukin-38 is released from apoptotic cells to limit inflammatory macrophage responses. J Mol Cell Biol.

[47] Takenaka S, Kaieda S, Kawayama T, Matsuokaa M, Kaku Y, Kinoshita T, Sakazaki Y, Okamoto M, Tominaga M, Kanesaki K (2015). IL-38: a new factor in rheumatoid arthritis. Biochem Biophys Rep.

[48] Boutet MA, Najm A, Bart G, Brion R, Touchais S, Trichet V, Layrolle P, Gabay C, Palmer G, Blanchard F, Le Goff B. IL-38 overexpression induces anti-inflammatory effects in mice arthritis models and in human macrophages in vitro. Ann Rheum Dis. 2017; 10.1136/annrheumdis-2016-210630. 10.1136/annrheumdis-2016-210630 28288964

[49] Wang M (2016). Wang B2, ma Z2, sun X2, tang Y2, Li X2, Wu X3. Detection of the novel IL-1 family cytokines by QAH-IL1F-1 assay in rheumatoid arthritis. Cell Mol Biol (Noisy-le-grand).

[50] Kumar S, Hanning CR, Brigham-Burke MR, Rieman DJ, Lehr R, Khandekar S, Kirkpatrick RB, Scott GF, Lee JC, Lynch FJ, Gao W, Gambotto A, Lotze MT (2002). Interleukin-1F7B (IL-1H4/IL-1F7) is processed by caspase-1 and mature IL-1F7B binds to the IL-18 receptor but does not induce IFN-gamma production. Cytokine.

[51] Bufler P, Gamboni-Robertson F, Azam T, Kim SH, Dinarello CA (2004). Interleukin-1 homologues IL-1F7b and IL-18 contain functional mRNA instability elements within the coding region responsive to lipopolysaccharide. Biochem J.

[52] Li S, Neff CP, Barber K, Hong J, Luo Y, Azam T, Palmer BE, Fujita M, Garlanda C, Mantovani A, Kim S, Dinarello CA (2015). Extracellular forms of IL-37 inhibit innate inflammation in vitro and in vivo but require the IL-1 family decoy receptor IL-1R8. Proc Natl Acad Sci U S A.

[53] Nold MF, Nold-Petry CA, Zepp JA, Palmer BE, Bufler P, Dinarello CA (2010). IL-37 is a fundamental inhibitor of innate immunity. Nat Immunol.

[54] Nold-Petry CA, Lo CY, Rudloff I, Elgass KD, Li S, Gantier MP, Lotz-Havla AS, Gersting SW, Cho SX, Lao JC, Ellisdon AM, Rotter B, Azam T, Mangan NE, Rossello FJ, Whisstock JC, Bufler P, Garlanda C, Mantovani A, Dinarello CA, Nold MF (2015). IL-37 requires the receptors IL-18Rα and IL-1R8 (SIGIRR) to carry out its multifaceted anti-inflammatory program upon innate signal transduction. Nat Immunol.

[55] McNamee EN, Masterson JC, Jedlicka P, McManus M, Grenz A, Collins CB, Nold MF, Nold-Petry C, Bufler P, Dinarello CA, Rivera-Nieves J (2011). Interleukin 37 expression protects mice from colitis. Proc Natl Acad Sci U S A.

[56] Teng X, Hu Z, Wei X, Wang Z, Guan T, Liu N, Liu X, Ye N, Deng G, Luo C, Huang N, Sun C, Xu M, Zhou X, Deng H, Edwards CK, Chen X, Wang X, Cui K, Wei Y, Li J (2014). IL-37 ameliorates the inflammatory process in psoriasis by suppressing proinflammatory cytokine production. J Immunol.

[57] Xu WD, Zhao Y, Liu Y (2015). Insights into IL-37, the role in autoimmune diseases. Autoimmun Rev.

[58] Weidlich S, Bulau AM, Schwerd T, Althans J, Kappler R, Koletzko S, Mayr D, Bufler P (2014). Intestinal expression of the anti-inflammatory interleukin-1 homologue IL-37 in pediatric inflammatory bowel disease. J Pediatr Gastroenterol Nutr.

[59] Ye L, Ji L, Wen Z, Zhou Y, Hu D, Li Y, Yu T, Chen B, Zhang J, Ding L, Du J, Huang Z (2014). IL-37 inhibits the production of inflammatory cytokines in peripheral blood mononuclear cells of patients with systemic lupus erythematosus: its correlation with disease activity. J Transl Med.

[60] Xia S, Wei J, Wang J, Sun H, Zheng W, Li Y, Sun Y, Zhao H, Zhang S, Wen T, Zhou X, Gao JX, Wang P, Wu Z, Zhao L, Yin Z (2014). A requirement of dendritic cell-derived interleukin-27 for the tumor infiltration of regulatory T cells. J Leukoc Biol.

[61] Chen B, Huang K, Ye L, Li Y, Zhang J, Zhang J, Fan X, Liu X, Li L, Sun J, Du J, Huang Z (2015). Interleukin-37 is increased in ankylosing spondylitis patients and associated with disease activity. Transl Med.

[62] Cavalli G, Koenders M, Kalabokis V, Kim J, Tan AC, Garlanda C, Mantovani A, Dagna L, Joosten LA, Dinarello CA (2016). Treating experimental arthritis with the innate immune inhibitor interleukin-37 reduces joint and systemic inflammation. Rheumatology (Oxford).

[63] Ye L, Jiang B, Deng J, Du J, Xiong W, Guan Y, Wen Z, Huang K, Huang Z (2015). IL-37 alleviates rheumatoid arthritis by suppressing IL-17 and IL-17-triggering cytokine production and limiting Th17 cell proliferation. J Immunol.

[64] Zhao PW, Jiang WG, Wang L, Jiang ZY, Shan YX, Jiang YF (2014). Plasma levels of IL-37 and correlation with TNF-α, IL-17A, and disease activity during DMARD treatment of rheumatoid arthritis. PLoS One.

[65] Xia T, Zheng XF, Qian BH, Fang H, Wang JJ, Zhang LL, Pang YF, Zhang J, Wei XQ, Xia ZF, Zhao DB (2015). Plasma interleukin-37 is elevated in patients with rheumatoid arthritis: its correlation with disease activity and Th1/Th2/Th17-related cytokines. Dis Markers.

[66] Xia L, Shen H, Lu J (2015). Elevated serum and synovial fluid levels of interleukin-37 in patients with rheumatoid arthritis: attenuated the production of inflammatory cytokines. Cytokine.

[67] Yang L, Zhang J, Tao J, Lu T (2015). Elevated serum levels of Interleukin-37 are associated with inflammatory cytokines and disease activity in rheumatoid arthritis. APMIS.

[68] Akdis M, Burgler S, Crameri R, Eiwegger T, Fujita H, Gomez E, Klunker S, Meyer N, O'Mahony L, Palomares O, Rhyner C, Ouaked N, Schaffartzik A, Van De Veen W, Zeller S, Zimmermann M, Akdis CA (2011). Interleukins, from 1 to 37, and interferon-g: receptors, functions, and roles in diseases. J Allergy Clin Immunol.

[69] Vignali DA, Kuchroo VK (2012). IL-12 family cytokines: immunological playmakers. Nat Immunol.

[70] Wang X, Wei Y, Xiao H, Liu X, Zhang Y, Han G, Chen G, Hou C, Ma N, Shen B, Li Y, Egwuagu CE, Wang R (2016). A novel IL-23p19/Ebi3 (IL-39) cytokine mediates inflammation in lupus-like mice. Eur J Immunol.

[71] Wang X, Liu X, Zhang Y, Wang Z, Zhu G, Han G, Chen G, Hou C, Wang T, Ma N, Shen B, Li Y, Xiao H, Wang R (2016). Interleukin (IL)-39 [IL-23p19/Epstein-Barr virus-induced 3 (Ebi3)] induces differentiation/expansion of neutrophils in lupus-prone mice. Clin Exp Immunol.

[72] Sawant DV, Hamilton K, Vignali DA (2015). Interleukin-35: expanding its job profile. J Interf Cytokine Res.

[73] Collison LW, Delgoffe GM, Guy CS, Vignali KM, Chaturvedi V, Fairweather D, Satoskar AR, Garcia KC, Hunter CA, Drake CG, Murray PJ, Vignali DA (2012). The composition and signaling of the IL-35 receptor are unconventional. Nat Immunol.

[74] Oppmann B, Lesley R, Blom B, Timans JC, Xu Y, Hunte B, Vega F, Yu N, Wang J, Singh K, Zonin F, Vaisberg E, Churakova T, Liu M, Gorman D, Wagner J, Zurawski S, Liu Y, Abrams JS, Moore KW, Rennick D, de Waal-Malefyt R, Hannum C, Bazan JF, Kastelein RA (2000). Novel p19 protein engages IL-12p40 to form a cytokine, IL-23, with biological activities similar as well as distinct from IL-12. Immunity.

[75] Pflanz S, Timans JC, Cheung J, Rosales R, Kanzler H, Gilbert J, Hibbert L, Churakova T, Travis M, Vaisberg E, Blumenschein WM, Mattson JD, Wagner JL, To W, Zurawski S, McClanahan TK, Gorman DM, Bazan JF, de Waal Malefyt R, Rennick D, Kastelein RA. IL-27, a heterodimeric cytokine composed of EBI3 and p28 protein, induces proliferation of naive CD4 + T cells. Immunity 2002;16(6):779–790. 10.1016/s1074-7613(02)00324-2 12121660

[76] Villarino AV, Stumhofer JS, Saris CJ, Kastelein RA, de Sauvage FJ, Hunter CA (2006). IL-27 limits IL-2 production during Th1 differentiation. J Immunol.

[77] Lucas S, Ghilardi N, Li J, de Sauvage FJ (2003). IL-27 regulates IL-12 responsiveness of naive CD4+ T cells through Stat1-dependent and -independent mechanisms. Proc Natl Acad Sci U S A.

[78] Villarino AV, Larkin J, Saris CJ, Caton AJ, Lucas S, Wong T, de Sauvage FJ, Hunter CA (2005). Positive and negative regulation of the IL-27 receptor during lymphoid cell activation. J Immunol.

[79] Batten M, Li J, Yi S, Kljavin NM, Danilenko DM, Lucas S, Lee J, de Sauvage FJ, Ghilardi N (2006). Interleukin 27 limits autoimmune encephalomyelitis by suppressing the development of interleukin 17-producing T cells. Nat Immunol.

[80] Sander LE, Obermeier F, Dierssen U, Kroy DC, Singh AK, Seidler U, Streetz KL, Lutz HH, Müller W, Tacke F, Trautwein C (2008). Gp130 signaling promotes development of acute experimental colitis by facilitating early neutrophil/macrophage recruitment and activation. J Immunol.

[81] Honda K, Nakamura K, Matsui N, Takahashi M, Kitamura Y, Mizutani T, Harada N, Nawata H, Hamano S, Yoshida H (2005). T helper 1-inducing property of IL-27/WSX-1 signaling is required for the induction of experimental colitis. Inflamm Bowel Dis.

[82] Jones GW, Bombardieri M, Greenhill CJ, McLeod L, Nerviani A, Rocher-Ros V, Cardus A, Williams AS, Pitzalis C, Jenkins BJ, Jones SA (2015). Interleukin-27 inhibits ectopic lymphoid-like structure development in early inflammatory arthritis. J Exp Med.

[83] Cao Y, Doodes PD, Glant TT, Finnegan A (2008). IL-27 induces a Th1 immune response and susceptibility to experimental arthritis. J Immunol.

[84] Park JS, Jung YO, Oh HJ, Park SJ, Heo YJ, Kang CM, Kwok SK, Ju JH, Park KS, Cho ML, Sung YC, Park SH, Kim HY (2012). Interleukin-27 suppresses osteoclastogenesis via induction of interferon-γ. Immunology.

[85] Pickens SR, Chamberlain ND, Volin MV, Mandelin AM, Agrawal H, Matsui M, Yoshimoto T, Shahrara S (2011). Local expression of interleukin-27 ameliorates collagen-induced arthritis. Arthritis Rheum.

[86] Niedbala W, Cai B, Wei X, Patakas A, Leung BP, McInnes IB, Liew FY (2008). Interleukin 27 attenuates collagen-induced arthritis. Ann Rheum Dis.

[87] Rajaiah R, Puttabyatappa M, Polumuri SK, Moudgil KD (2011). Interleukin-27 and interferon-gamma are involved in regulation of autoimmune arthritis. J Biol Chem.

[88] Shen H, Xia L, Xiao W, Lu J (2011). Increased levels of interleukin-27 in patients with rheumatoid arthritis. Arthritis Rheum.

[89] Wong CK, Chen DP, Tam LS, Li EK, Yin YB, Lam CW (2010). Effects of inflammatory cytokine IL-27 on the activation of fibroblast-like synoviocytes in rheumatoid arthritis. Arthritis Res Ther.

[90] Tanida S, Yoshitomi H, Ishikawa M, Kasahara T, Murata K, Shibuya H, Ito H, Nakamura T (2011). IL-27-producing CD14(+) cells infiltrate inflamed joints of rheumatoid arthritis and regulate inflammation and chemotactic migration. Cytokine.

[91] Choi J, Leung PS, Bowlus C, Gershwin ME (2015). IL-35 and autoimmunity: a comprehensive perspective. Clinic Rev Allerg Immunol.

[92] Collison LW, Delgoffe GM, Guy CS, Vignali KM, Chaturvedi V, Fairweather D, Satoskar AR, Garcia KC, Hunter CA, Drake CG, Murray PJ (2012). Vignali DA the composition and signaling of the IL-35 receptor are unconventional. Nat Immunol.

[93] Shen P, Roch T, Lampropoulou V, O'Connor RA, Stervbo U, Hilgenberg E, Ries S, Dang VD, Jaimes Y, Daridon C, Li R, Jouneau L, Boudinot P, Wilantri S, Sakwa I, Miyazaki Y, Leech MD, McPherson RC, Wirtz S, Neurath M, Hoehlig K, Meinl E, Grützkau A, Grün JR, Horn K, Kühl AA, Dörner T, Bar-Or A, Kaufmann SH, Anderton SM, Fillatreau S (2014). IL-35-producing B cells are critical regulators of immunity during autoimmune and infectious diseases. Nature.

[94] Collison LW, Workman CJ, Kuo TT, Boyd K, Wang Y, Vignali KM, Cross R, Sehy D, Blumberg RS, Vignali DA (2007). The inhibitory cytokine IL-35 contributes to regulatory T-cell function. Nature.

[95] Wang RX, Yu CR, Dambuza IM, Mahdi RM, Dolinska MB, Sergeev YV, Wingfield PT, Kim SH, Egwuagu CE (2014). Interleukin-35 induces regulatory B cells that suppress autoimmune disease. Nat Med.

[96] Collison LW, Chaturvedi V, Henderson AL, Giacomin PR, Guy C, Bankoti J, Finkelstein D, Forbes K, Workman CJ, Brown SA, Rehg JE, Jones ML, Ni HT, Artis D, Turk MJ, Vignali DA (2010). IL-35-mediated induction of a potent regulatory T cell population. Nat Immunol.

[97] Marrelli A, Cipriani P, Liakouli V, Carubbi F, Perricone C, Perricone R, Giacomelli R (2011). Angiogenesis in rheumatoid arthritis: a disease specific process or a common response to chronic inflammation?. Autoimmun Rev.

[98] Niedbala W, Wei X, Cai B, Hueber AJ, Leung BP, IB MI, Liew FY (2007). IL-35 is a novel cytokine with therapeutic effects against collagen-induced arthritis through the expansion of regulatory T cells and suppression of Th17 cells. Eur J Immunol.

[99] Li Y, Wu S, Li Y, Jiang S, Lin T, Xia L, Shen H, Lu J (2016). Interleukin-35 (IL-35) inhibits proliferation and promotes apoptosis of fibroblast-like synoviocytes isolated from mice with collagen-induced arthritis. Mol Biol Rep.

[100] Wu S, Li Y, Li Y, Yao L, Lin T, Jiang S, Shen H, Xia L, Lu J (2016). Interleukin-35 attenuates collagen-induced arthritis through suppression of vascular endothelial growth factor and its receptors. Int Immunopharmacol.

[101] Šenolt L, Šumová B, Jandová R, Hulejová H, Mann H, Pavelka K, Vencovský J, Filková M (2015). Interleukin 35 synovial fluid levels are associated with disease activity of rheumatoid arthritis. PLoS One.

[102] Filková M, Vernerová Z, Hulejová H, Prajzlerová K, Veigl D, Pavelka K, Vencovský J, Šenolt L (2015). Pro-inflammatory effects of interleukin-35 in rheumatoid arthritis. Cytokine.

[103] Nakano S, Morimoto S, Suzuki S, Tsushima H, Yamanaka K, Sekigawa I, Takasaki Y (2015). Immunoregulatory role of IL-35 in T cells of patients with rheumatoid arthritis. Rheumatology (Oxford).

[104] Kim SH, Han SY, Azam T, Yoon DY, Dinarello CA (2005). Interleukin-32: a cytokine and inducer of TNFalpha. Immunity.

[105] Kang JW, Park YS, Lee DH, Kim MS, Bak Y, Ham SY, Park SH, Kim H, Ahn JH, Hong JT, Yoon DY (2014). Interaction network mapping among IL-32 isoforms. Biochimie.

[106] Choi JD, Bae SY, Hong JW, Azam T, Dinarello CA, Her E, Choi WS, Kim BK, Lee CK, Yoon DY, Kim SJ, Kim SH (2009). Identification of the most active interleukin-32 isoform. Immunology.

[107] Khawar MB, Abbasi M, Sheikh N (2016). IL-32: a novel pluripotent inflammatory interleukin, towards gastric inflammation, gastric cancer, and chronic rhino sinusitis. Mediat Inflamm.

[108] Dahl CA, Schall RP, He HL, Cairns JS (1992). Identification of a novel gene expressed in activated natural killer cells and T cells. J Immunol.

[109] Ribeiro-Dias F, Saar Gomes R, de Lima Silva LL, Dos Santos JC, Joosten LA (2017). Interleukin 32: a novel player in the control of infectious diseases. J Leukoc Biol.

[110] Heinhuis B, Koenders MI, van de Loo FA, Netea MG, van den Berg WB, Joosten LA (2011). Inflammation-dependent secretion and splicing of IL-32g in rheumatoid arthritis. Proc Natl Acad Sci U S A.

[111] Kim SH, Han SY, Azam T, Yoon DY, Dinarello CA (2005). Interleukin-32: a cytokine and inducer of TNFa. Immunity.

[112] Hasegawa H, Thomas HJ, Schooley K, Born TL (2011). Native IL-32 is released from intestinal epithelial cells via a non-classical secretory pathway as a membrane-associated protein. Cytokine.

[113] Khawar MB, Abbasi M, Sheikh N (2015). A panoramic spectrum of complex interplay between the immune system and IL-32 during pathogenesis of various systemic infections and inflammation. Eur J Med Res.

[114] Netea MG, Lewis EC, Azam T, Joosten LA, Jaekal J, Bae SY, Dinarello CA, Kim SH (2008). Interleukin-32 induces the differentiation of monocytes into macrophage-like cells. Proc Natl Acad Sci U S A.

[115] Jung MY, Son MH, Kim SH, Cho D, Kim TS (2011). IL-32gamma induces the maturation of dendritic cells with Th1- and Th17-polarizing ability through enhanced IL-12 and IL-6 production. J Immunol.

[116] Netea MG, Azam T, Ferwerda G, Girardin SE, Walsh M, Park JS, Abraham E, Kim JM, Yoon DY, Dinarello CA, Kim SH (2005). IL-32 synergizes with nucleotide oligomerization domain (NOD) 1 and NOD2 ligands for IL-1beta and IL-6 production through a caspase 1-dependent mechanism. Proc Natl Acad Sci U S A.

[117] Kobayashi H, Huang J, Ye F, Shyr Y, Blackwell TS, Lin PC (2010). Interleukin-32beta propagates vascular inflammation and exacerbates sepsis in a mouse model. PLoS One.

[118] Dinarello CA, Kim SH (2006). IL-32, a novel cytokine with a possible role in disease. Ann Rheum Dis.

[119] Park YE, Kim GT, Lee SG, Park SH, Baek SH, Kim SI, Kim JI, Jin HS (2013). IL-32 aggravates synovial inflammation and bone destruction and increases synovial natural killer cells in experimental arthritis models. Rheumatol Int.

[120] Nakayama M, Niki Y, Kawasaki T, Takeda Y, Horiuchi K, Sasaki A, Okada Y, Umezawa K, Ikegami H, Toyama Y, Miyamoto T (2012). Enhanced susceptibility to lipopolysaccharide-induced arthritis and endotoxin shock in interleukin-32 alpha transgenic mice through induction of tumor necrosis factor alpha. Arthritis Res Ther.

[121] Cagnard N, Letourneur F, Essabbani A, Devauchelle V, Mistou S, Rapinat A, Decraene C, Fournier C, Chiocchia G (2005). Interleukin-32, CCL2, PF4F1 and GFD10 are the only cytokine/chemokine genes differentially expressed by in vitro cultured rheumatoid and osteoarthritis fibroblast-like synoviocytes. Eur Cytokine Netw.

[122] Xu WD, Zhang M, Feng CC, Yang XK, Pan HF, Ye DQ (2013). IL-32 with potential insights into rheumatoid arthritis. Clin Immunol.

[123] Kim YG, Lee CK, Oh JS, Kim SH, Kim KA, Yoo B (2010). Effect of interleukin-32gamma on differentiation of osteoclasts from CD14+ monocytes. Arthritis Rheum.

[124] Moon YM, Yoon BY, Her YM, Oh HJ, Lee JS, Kim KW, Lee SY, Woo YJ, Park KS, Park SH, Kim HY, Cho ML (2012). IL-32 and IL-17 interact and have the potential to aggravate osteoclastogenesis in rheumatoid arthritis. Arthritis Res Ther.

[125] Müller-Ladner U, Ospelt C, Gay S, Distler O, Pap T (2007). Cells of the synovium in rheumatoid arthritis. Synovial fibroblasts Arthritis Res Ther.

[126] Alsaleh G, Sparsa L, Chatelus E, Ehlinger M, Gottenberg JE, Wachsmann D, Sibilia J (2010). Innate immunity triggers IL-32 expression by fibroblast-like synoviocytes in rheumatoid arthritis. Arthritis Res Ther.

[127] Mun SH, Kim JW, Nah SS, Ko NY, Lee JH, Kim JD, Kim DK, Kim HS, Choi JD, Kim SH, Lee CK, Park SH, Kim BK, Kim HS, Kim YM, Choi WS (2009). Tumor necrosis factor alpha-induced interleukin-32 is positively regulated via the Syk/protein kinase Cdelta/JNK pathway in rheumatoid synovial fibroblasts. Arthritis Rheum.

[128] Joosten LA, Netea MG, Kim SH, Yoon DY, Oppers-Walgreen B, Radstake TR, Barrera P, van de Loo FA, Dinarello CA, van den Berg WB (2006). IL-32, a proinflammatory cytokine in rheumatoid arthritis. Proc Natl Acad Sci U S A.

[129] Heinhuis B, Koenders MI, van Riel PL, van de Loo FA, Dinarello CA, Netea MG, van den Berg WB, Joosten LA (2011). Tumour necrosis factor alpha-driven IL-32 expression in rheumatoid arthritis synovial tissue amplifies an inflammatory cascade. Ann Rheum Dis.

[130] Gui M, Zhang H, Zhong K, Li Y, Sun J, Wang L (2013). Clinical significance of interleukin-32 expression in patients with rheumatoid arthritis. Asian Pac J Allergy Immunol.

[131] Lin H, Lee E, Hestir K, Leo C, Huang M, Bosch E, Halenbeck R, Wu G, Zhou A, Behrens D, Hollenbaugh D, Linnemann T, Qin M, Wong J, Chu K, Doberstein SK, Williams LT (2008). Discovery of a cytokine and its receptor by functional screening of the extracellular proteome. Science.

[132] Wang Y, Colonna M (2014). Interkeukin-34, a cytokine crucial for the differentiation and maintenance of tissue resident macrophages and Langerhans cells. Eur J Immunol.

[133] Nandi S, Cioce M, Yeung YG, Nieves E, Tesfa L, Lin H, Hsu AW, Halenbeck R, Cheng HY, Gokhan S, Mehler MF, Stanley ER (2013). Receptor-type protein-tyrosine phosphatase ζ is a functional receptor for interleukin-34. J Biol Chem.

[134] Segaliny AI, Brion R, Mortier E, Maillasson M, Cherel M, Jacques Y, Le Goff B, Heymann D (1853). Syndecan-1 regulates the biological activities of interleukin-34. Biochim Biophys Acta.

[135] Bézie S, Picarda E, Ossart J, Tesson L, Usal C, Renaudin K, Anegon I, Guillonneau C (2015). IL-34 is a Treg-specific cytokine and mediates transplant tolerance. J Clin Invest.

[136] Foucher ED, Blanchard S, Preisser L, Garo E, Ifrah N, Guardiola P, Delneste Y, Jeannin P (2013). IL-34 induces the differentiation of human monocytes into immunosuppressive macrophages. Antagonistic effects of GM-CSF and IFNγ. PLoS One.

[137] Chen Z, Buki K, Vääräniemi J, Gu G, Väänänen HK (2011). The critical role of IL-34 in osteoclastogenesis. PLoS One.

[138] Baud'huin M, Renault R, Charrier C, Riet A, Moreau A, Brion R, Gouin F, Duplomb L, Heymann D (2010). Interleukin-34 is expressed by giant cell tumours of bone and plays a key role in RANKL-induced osteoclastogenesis. J Pathol.

[139] Nakamichi Y, Mizoguchi T, Arai A, Kobayashi Y, Sato M, Penninger JM, Yasuda H, Kato S, DeLuca HF, Suda T, Udagawa N, Takahashi N (2012). Spleen serves as a reservoir of osteoclast precursors through vitamin D-induced IL-34 expression in osteopetrotic op/op mice. Proc Natl Acad Sci U S A.

[140] Wang Y, Szretter KJ, Vermi W, Gilfillan S, Rossini C, Cella M, Barrow AD, Diamond MS, Colonna M (2012). IL-34 is a tissue-restricted ligand of CSF1R required for the development of Langerhans cells and microglia. Nat Immunol.

[141] Greter M, Lelios I, Pelczar P, Hoeffel G, Price J, Leboeuf M, Kündig TM, Frei K, Ginhoux F, Merad M, Becher B (2012). Stroma-derived interleukin-34 controls the development and maintenance of langerhans cells and the maintenance of microglia. Immunity.

[142] Erblich B, Zhu L, Etgen AM, Dobrenis K, Pollard JW (2011). Absence of colony stimulation factor-1 receptor results in loss of microglia, disrupted brain development and olfactory deficits. PLoS One.

[143] Liddelow SA, Guttenplan KA, Clarke LE, Bennett FC, Bohlen CJ, Schirmer L, Bennett ML, Münch AE, Chung WS, Peterson TC, Wilton DK, Frouin A, Napier BA, Panicker N, Kumar M, Buckwalter MS, Rowitch DH, Dawson VL, Dawson TM, Stevens B, Barres BA (2017). Neurotoxic reactive astrocytes are induced by activated microglia. Nature.

[144] Dai XM, Ryan GR, Hapel AJ, Dominguez MG, Russell RG, Kapp S, Sylvestre V, Stanley ER (2002). Targeted disruption of the mouse colony-stimulating factor 1 receptor gene results in osteopetrosis, mononuclear phagocyte deficiency, increased primitive progenitor cell frequencies, and reproductive defects. Blood.

[145] Louis C, Cook AD, Lacey D, Fleetwood AJ, Vlahos R, Anderson GP, Hamilton JA (2015). Specific contributions of CSF-1 and GM-CSF to the dynamics of the mononuclear phagocyte system. J Immunol.

[146] Bischof RJ, Zafiropoulos D, Hamilton JA, Campbell IK (2000). Exacerbation of acute inflammatory arthritis by the colony-stimulating factors CSF-1 and granulocyte macrophage (GM)-CSF: evidence of macrophage infiltration and local proliferation. Clin Exp Immunol.

[147] Campbell IK, Rich MJ, Bischof RJ, Hamilton JA (2000). The colony-stimulating factors and collagen-induced arthritis: exacerbation of disease by M-CSF and G-CSF and requirement for endogenous M-CSF. J Leukoc Biol.

[148] Garcia S, Hartkamp LM, Malvar-Fernandez B, van Es IE, Lin H, Wong J, Long L, Zanghi JA, Rankin AL, Masteller EL, Wong BR, Radstake TR, Tak PP, Reedquist KA (2016). Colony-stimulating factor (CSF) 1 receptor blockade reduces inflammation in human and murine models of rheumatoid arthritis. Arthritis Res Ther.

[149] Moon SJ, Hong YS, Ju JH, Kwok SK, Park SH, Min JK (2013). Increased levels of interleukin 34 in serum and synovial fluid are associated with rheumatoid factor and anticyclic citrullinated peptide antibody titers in patients with rheumatoid arthritis. J Rheumatol.

[150] Zhang F, Ding R, Li P, Ma C, Song D, Wang X, Ma T, Bi L (2015). Interleukin-34 in rheumatoid arthritis: potential role in clinical therapy. Int J Clin Exp Med.

[151] Tian Y, Shen H, Xia L, Lu J (2013). Elevated serum and synovial fluid levels of interleukin-34 in rheumatoid arthritis: possible association with disease progression via interleukin-17 production. J Interf Cytokine Res.

[152] Chang SH, Choi BY, Choi J, Yoo JJ, Ha YJ, Cho HJ, Kang EH, Song YW, Lee YJ (2015). Baseline serum interleukin-34 levels independently predict radiographic progression in patients with rheumatoid arthritis. Rheumatol Int.

[153] Yang S, Jiang S, Wang Y, Tu S, Wang Z, Chen Z (2016). Interleukin 34 Upregulation contributes to the increment of MicroRNA 21 expression through STAT3 activation associated with disease activity in rheumatoid arthritis. J Rheumatol.

[154] Hwang SJ, Choi B, Kang SS, Chang JH, Kim YG, Chung YH, Sohn DH, So MW, Lee CK, Robinson WH, Chang EJ (2012). Interleukin-34 produced by human fibroblast-like synovial cells in rheumatoid arthritis supports osteoclastogenesis. Arthritis Res Ther.

[155] Chemel M, Le Goff B, Brion R, Cozic C, Berreur M, Amiaud J, Bougras G, Touchais S, Blanchard F, Heymann MF, Berthelot JM, Verrecchia F, Heymann D (2012). Interleukin 34 expression is associated with synovitis severity in rheumatoid arthritis patients. Ann Rheum Dis.

